# Increasing diagnoses per patient admission at a specialist children’s hospital: A retrospective study

**DOI:** 10.1371/journal.pone.0322997

**Published:** 2025-05-06

**Authors:** Stuart A. Bowyer, John Booth, Daniel Key, Eleni Pissaridou, William A. Bryant, Harry Hemingway, Neil J. Sebire

**Affiliations:** 1 Data Research, Innovation and Virtual Environments (DRIVE) Unit, Great Ormond Street Hospital, London United Kingdom; 2 NIHR GOSH Biomedical Research Centre, London, United Kingdom; 3 Department of Surgery and Cancer, Imperial College London, London, United Kingdom; 4 Institute of Health Informatics, Faculty of Population Health Sciences, University College London, London, United Kingdom; 5 NIHR University College London Hospitals NHS Trust Biomedical Research Centre, London, United Kingdom; 6 Health Data Research, London, United Kingdom; 7 Population, Policy and Practice Research and Teaching Department, UCL Great Ormond Street Institute of Child Health, London, United Kingdom; University of Oxford, UNITED KINGDOM OF GREAT BRITAIN AND NORTHERN IRELAND

## Abstract

**Objective:**

In adult practice there is recognition that average patient complexity is increasing, with a greater proportion of patients having multiple diagnoses or comorbidities. This study aims to examine whether there has been a change in number of recorded coexisting diagnoses per patient over a 24-year period for children attending as in-patients to a specialist children’s hospital in England.

**Methods:**

Following all in-patient admissions, patient episodes are allocated specific diagnosis codes (ICD-10) by a specialist clinical coding team according to standard NHS criteria and guidance. We examine the number of coexisting diagnoses allocated per patient admission over a 24-year period.

**Results:**

From a total of 278,579 overnight in-patient admissions during the study period (2000–2023) there were 1,023,276 ICD-10 patient diagnoses. The mean number of diagnoses per admission increased from 2.72 to 10.43 over the period (Kendall’s tau statistic of 0.93; p-value < 0.001), an increase of 284% (95% confidence interval 275% - 293%).

**Conclusions:**

Over recent decades, the recorded complexity of patients attending a specialist children’s hospital appear to have increased significantly, with an almost 3-fold increase in the number of coexisting diagnoses present per admission. The cause of this finding cannot be determined from the data; however, it appears to be gradual and consistent, and across all speciality areas suggesting biological or referral factors rather than artefactual coding issues. Recognition of such a trend is important when interpreting retrospective data for AI, research, and planning purposes.

## Introduction

Understanding changing patterns in the co-occurrence and reporting of disease in children is fundamental to the development of appropriate services, and the design and interpretation of clinical trials and other research. Patients with high comorbidity are known to require more from the healthcare providers in terms of cost and complexity of care [1] and are routinely excluded from clinical trials [2]. Based on data from adults, it has been suggested that patients are becoming more complex, with increasing numbers of comorbidities and complex chronic conditions. For example, in the United States, > 85% of healthcare spending is related to patients with one or more chronic conditions, and more than half of physician practices regularly see patients with three or more comorbidities [3].

However, the temporal trends in disease co-occurrence in children are unclear. Previous studies have found both an increasing percentage of children hospitalised with specific complex or multi-system chronic conditions [4,5], and an increasing prevalence of children with multiple complex chronic conditions in the population [6], purportedly as the result of improving survival of childhood-onset disease [7]. However, these studies focus on chronic conditions and do not study the full range of chronic and acute co-occurring diagnoses observed in children. Furthermore, previous studies do not report the differing trends across diagnosis categories. Essentially the *only* way to study this is with Electronic Health Records (EHR).

The coding and subsequent reimbursement for clinical activity is a critical purpose for diagnoses in EHR data that can have substantial financial implications for a healthcare provider. As a result, diagnosis coding is subject to strict rules and guidance [8] which is changed and updated over time. Additionally, one of the rationales for establishing tertiary referral centres, such as the Great Ormond Street Hospital (GOSH) where this study was performed, is the ability to manage complex patients. Therefore, understanding what constitutes complex patients is important to wider healthcare service planning.

Therefore, the objectives of this study were to explore the numbers of all primary and secondary coexisting diagnoses recorded for each patient admission at a specialist children’s hospital over a 24-year period to determine whether similar trends are also occurring in tertiary care paediatric practice.

## Methods

### Cohort and data

To study the trends in diagnoses number and type, we examined diagnosis and admissions data for all inpatient admissions to a specialist UK children’s hospital, GOSH, between 1^st^ January 2000 and 31^st^ December 2023. GOSH is a highly specialist paediatric hospital that sees approximately 280,000 inpatient and outpatient visits per year. Diagnosis and admissions data for all patients since May 2019 were retrieved from the hospital electronic health record system, while previous data were available in the data warehouse created from administrative data available by the GOSH Digital Research Environment (DRE). Data were routinely de-identified on extraction by the standard processes through the DRE with analysis performed using only deidentified data in a secure trusted research environment within the NHS organisation [9]. Data were accessed for this study on 8^th^ October 2024.

Admissions data define the start and end date of all admissions. Only overnight inpatient admissions, i.e., covering two or more days and excluding day cases, were considered for the analysis due to changes in coding practice for other admission types. All clinical coding diagnoses, not restricted to primary diagnoses, associated with an included inpatient admission were considered for the analysis. Diagnosis data were coded at the subcategory level using ICD-10 (e.g., A01.1) by the clinical coding team at the end of the admission according to standard guidelines. Diagnoses allocated or mentioned by clinical and nursing teams directly within the patient record narrative or problem list were not examined as part of this study; only the final diagnoses recorded by the clinical coding team were used to improve consistency of recording and minimise variation between specialities or teams.

### Statistical analysis

The primary metric assessed in this study was the number of diagnoses per admission per year. To calculate this value, every overnight admission was first identified. All clinical coding diagnoses associated with each admission were retrieved and the number of unique diagnoses per patient admission were calculated. Repeated diagnoses of the same ICD-10 code, either contiguous or distinct, within an admission were only counted once for this analysis since we were interested in number of coexisting diagnoses. In the case of patients with multiple admissions in a year, the median value of unique diagnoses per admission across all their admissions that year was used.

For each calendar year, we calculated the mean number of diagnoses per patient admission as well as distributions plots. The change in mean between the start and end of the analysis period was computed from the difference in means and confidence intervals were computed using bootstrapping with 1000 replicas.

Diagnosis rates were stratified by ICD-10 chapter to explore individual trends. We analysed all 22 chapters (top-level) from the ICD-10:2019 hierarchy. For each stratum, the mean number of diagnoses per chapter per admission was calculated.

The statistical significance of trend changes over the study period were assessed using the Mann-Kendall trend test [10]. For this test, Kendall’s tau statistic was used to quantify the strength and direction of trend, and two-sided p-values were produced. The resulting p-values were adjusted for multiple comparisons by the Holm method [11].

Diagnosis rates trends were also stratified by patient age group, and number of previous admissions and visualised.

All analysis was performed using the R programming language.

This study was approved by the London - South East Research Ethics Committee, under reference number 21/LO/0646. The research involved the use of de-identified data and therefore the authors did not have access to information that could identify individual participants. As such, in accordance with ethical guidelines and institutional policies, informed consent was not required.

## Results

During the period of study, data were available from a total of 278,579 overnight admissions across 119,957 patients, representing 1,023,276 unique ICD-10 patient diagnosis codes (excluding repeats). Table 1 shows the demographic characteristics for the patient cohort whose admissions were analysed.

**Table 1:** Demographic characteristics of the patient population.

Characteristic	All 2000–2023, N = 119,957^1^	Year 2000, N = 7,129^1^	Year 2023, N = 7,885^1^
Age at first admission (years)	3.1 (0.6, 8.8)	4.6 (1.2, 10.0)	5.9 (1.9, 11.5)
Sex			
Female	52,616 (44%)	3,085 (43%)	3,444 (44%)
Male	67,335 (56%)	4,044 (57%)	4,441 (56%)
Indeterminate/Unspecified	6 (<0.1%)	0 (0%)	0 (0%)
Ethnicity			
White	59,974 (50%)	4,772 (67%)	3,693 (47%)
Mixed	3,523 (2.9%)	22 (0.3%)	420 (5.3%)
Asian or Asian British	12,912 (11%)	636 (8.9%)	1,124 (14%)
Black or Black British	8,681 (7.2%)	539 (7.6%)	582 (7.4%)
Other Ethnic Groups	10,030 (8.4%)	622 (8.7%)	913 (12%)
Unknown	24,837 (21%)	538 (7.5%)	1,153 (15%)

^1^Median (IQR); n (%).

**Fig 1:**
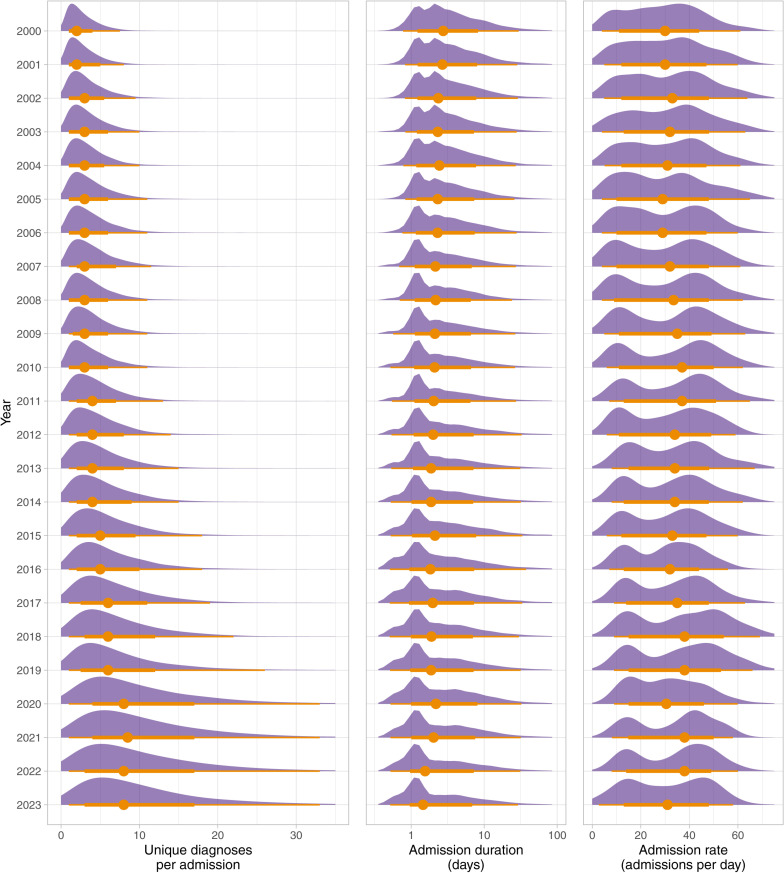
Annual distributions of diagnoses per admission between 2000 and 2023. The left-hand panel shows annual distributions of unique diagnoses per admission. The middle panel shows the annual distributions of admission durations, in days. The right-hand panel shows the annual distributions of daily admission rate. All plots show the normalised density plots, annual median value (circle), as well as intervals (lines).

The yearly distributions for number of unique diagnoses per admission, number of admissions per day and admission duration are shown in Fig 1. Fig 1 shows the overall mean diagnosis rate significantly increased from 2.72 diagnoses per admission in 2000 to 10.43 diagnoses per admission in 2023, an increase of 284% (95% confidence interval 275%-293%). The Mann-Kendall trend test showed a significant strong positive trend in diagnoses per admission with a Kendall’s tau statistic of 0.93 (p-value < 0.001).

The overall number of annual admissions increased from 10,659 in 2000 to 10,847 in 2023. The median admission duration reduced from 2.81 days (IQR 1.31–6.12) in 2000 to 1.49 days (IQR 1.09–4.93) in 2023.

**Fig 2:**
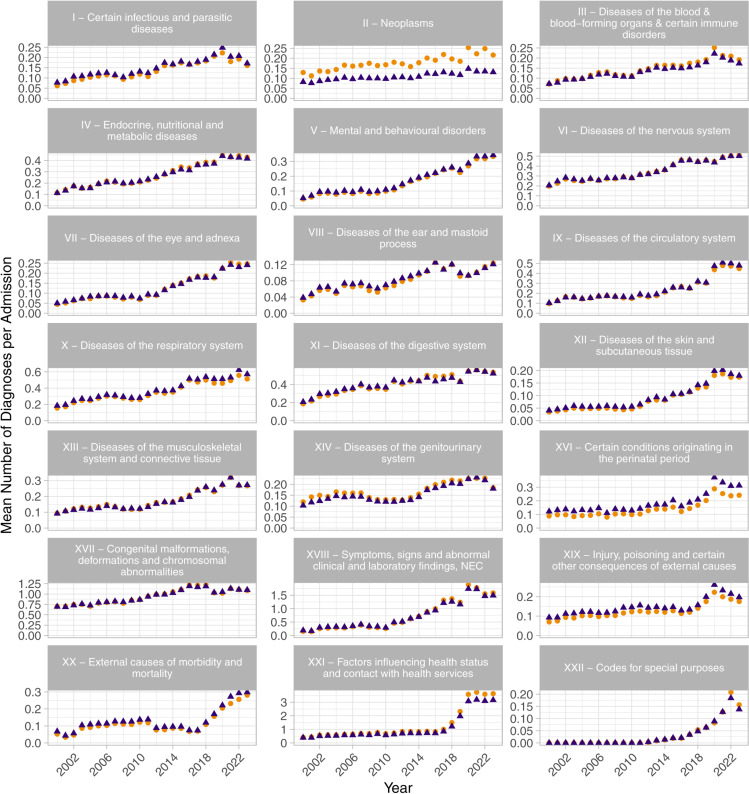
Yearly mean number of diagnoses per admission, stratified by ICD-10 chapter, for the chapters of ICD-10, excluding “XV - Pregnancy, childbirth and the puerperium”. Blue triangles show the mean annual diagnoses per admission, and orange circles show the mean annual diagnoses per admission with repeated admissions for an individual patient included.

The change in diagnosis rate between 2000 and 2023, stratified by chapters of ICD-10, are shown in Table 2. For those chapters with diagnoses in 2000, the chapter diagnosis rates increased by between 58% and 713%, with the majority showing significant strong positive trends based on the Mann-Kendall trend tests. The yearly mean number of diagnoses per admission, by ICD-10 chapter, are shown in Fig 2.

**Table 2:** Comparison of the mean number of unique diagnoses per hospital admission, between 2000 and 2023, stratified by chapters of ICD-10. The table shows absolute and relative changes in the number of diagnoses, by chapter, from a total of 2.72 diagnoses per admission in 2000 to 10.43 diagnoses per admission in 2023, an increase of 284% (275%, 293%). The table also shows 95% confidence intervals and the results of a Mann-Kendall (MK) trend test as a Kendall’s tau statistic and associated two-sided p-value to identify consistently increasing or decreasing trends. All p-values have been adjusted for multiple comparisons by the Holm method.

ICD-10 Chapter	2000	2023	Absolute Increase (95% CI)	Relative Increase (95% CI)	MK Tau (p-value)
I - Certain infectious and parasitic diseases (A00-B99)	0.08	0.17	0.09 (0.06, 0.08)	122% (96%, 159%)	0.79 (< 0.001)
II - Neoplasms (C00-D48)	0.08	0.13	0.05 (0.04, 0.06)	60% (42%, 77%)	0.77 (< 0.001)
III - Diseases of the blood & blood-forming organs & certain immune disorders (D50-D89)	0.07	0.17	0.10 (0.07, 0.09)	141% (95%, 148%)	0.83 (< 0.001)
IV - Endocrine, nutritional and metabolic diseases (E00-E90)	0.11	0.42	0.31 (0.26, 0.30)	271% (232%, 296%)	0.90 (< 0.001)
V - Mental and behavioural disorders (F00-F99)	0.05	0.34	0.29 (0.27, 0.30)	561% (510%, 679%)	0.91 (< 0.001)
VI - Diseases of the nervous system (G00-G99)	0.21	0.50	0.30 (0.27, 0.31)	145% (133%, 168%)	0.91 (< 0.001)
VII - Diseases of the eye and adnexa (H00-H59)	0.05	0.24	0.19 (0.17, 0.20)	384% (357%, 502%)	0.83 (< 0.001)
VIII - Diseases of the ear and mastoid process (H60-H95)	0.04	0.12	0.08 (0.07, 0.09)	216% (196%, 296%)	0.72 (< 0.001)
IX - Diseases of the circulatory system (I00-I99)	0.10	0.48	0.38 (0.33, 0.39)	367% (353%, 456%)	0.77 (< 0.001)
X - Diseases of the respiratory system (J00-J99)	0.18	0.57	0.39 (0.34, 0.38)	212% (208%, 258%)	0.81 (< 0.001)
XI - Diseases of the digestive system (K00-K93)	0.21	0.52	0.32 (0.27, 0.32)	153% (145%, 184%)	0.86 (< 0.001)
XII - Diseases of the skin and subcutaneous tissue (L00-L99)	0.04	0.18	0.14 (0.10, 0.12)	343% (253%, 370%)	0.82 (< 0.001)
XIII - Diseases of the musculoskeletal system and connective tissue (M00-M99)	0.09	0.27	0.18 (0.15, 0.19)	196% (172%, 233%)	0.78 (< 0.001)
XIV - Diseases of the genitourinary system (N00-N99)	0.10	0.18	0.08 (0.06, 0.08)	75% (55%, 93%)	0.51 (0.041)
XV - Pregnancy, childbirth and the puerperium (O00-O99)	0.00	0.00	0.00 (0.00, 0.00)		0.24 (1)
XVI - Certain conditions originating in the perinatal period (P00-P96)	0.12	0.31	0.19 (0.14, 0.21)	157% (123%, 212%)	0.77 (< 0.001)
XVII - Congenital malformations, deformations and chromosomal abnormalities (Q00-Q99)	0.70	1.10	0.40 (0.36, 0.45)	58% (51%, 67%)	0.78 (< 0.001)
XVIII - Symptoms, signs and abnormal clinical and laboratory findings, NEC (R00-R99)	0.19	1.49	1.30 (1.21, 1.29)	685% (708%, 828%)	0.85 (< 0.001)
XIX - Injury, poisoning and certain other consequences of external causes (S00-T98)	0.09	0.20	0.10 (0.06, 0.09)	113% (78%, 135%)	0.78 (< 0.001)
XX - External causes of morbidity and mortality (V01-Y98)	0.07	0.30	0.23 (0.18, 0.20)	341% (330%, 436%)	0.48 (0.083)
XXI - Factors influencing health status and contact with health services (Z00-Z99)	0.39	3.16	2.77 (2.72, 2.86)	713% (759%, 843%)	0.90 (< 0.001)
XXII - Codes for special purposes (U00-U85)	0.00	0.14	0.14 (0.11, 0.13)		0.89 (< 0.001)
Total (A00-Z99)	2.72	10.43	7.72 (7.52, 7.90)	284% (275%, 293%)	0.93 (< 0.001)

**Fig 3:**
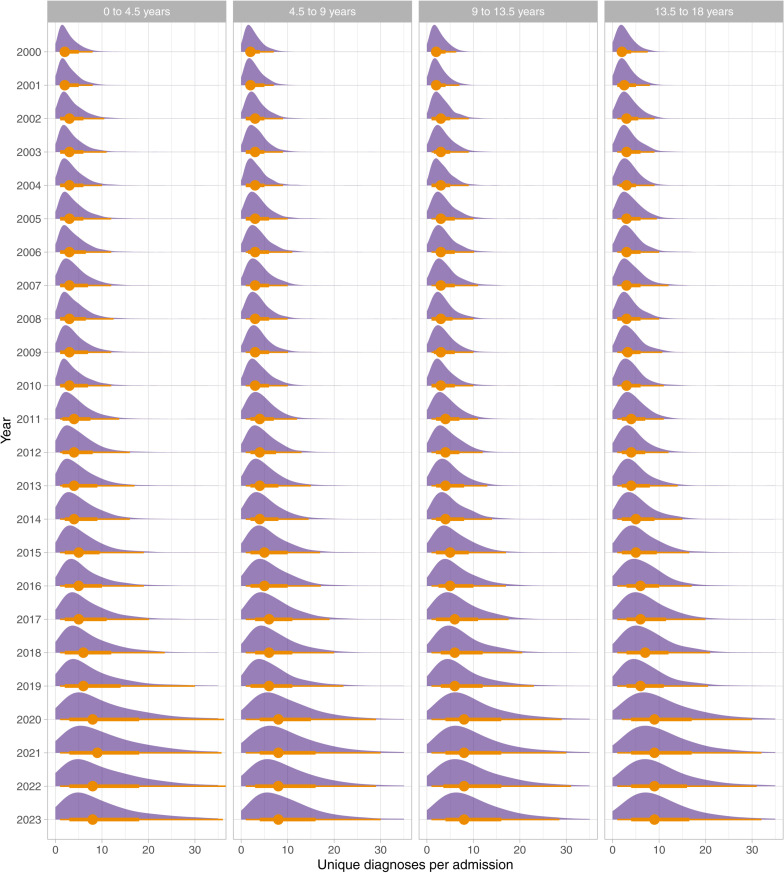
Annual distributions of diagnoses per admission between 2000 and 2023 stratified by age group. The plots show the normalised density plots, annual median value (circle), as well as intervals (lines).

Fig 3 shows the annual distributions of number of diagnoses per admission stratified by age group.

**Fig 4:**
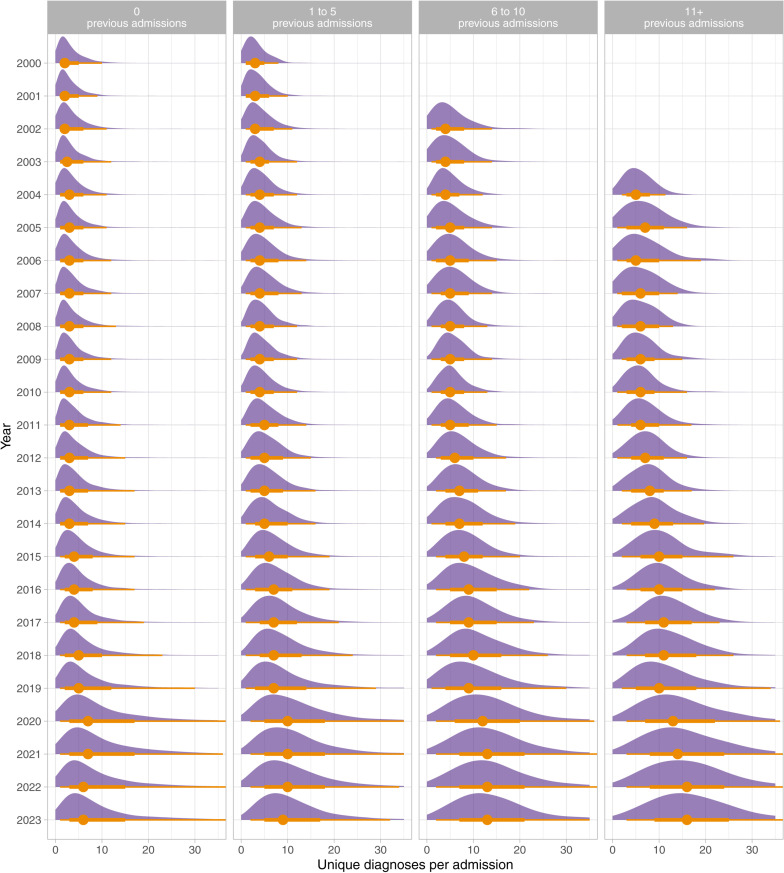
Annual distributions of diagnoses per admission between 2000 and 2023 stratified by the total number of previous life-time admissions to the hospital, for all patients born since the year 2000. The plots show the normalised density plots, annual median value (circle), as well as intervals (lines). Periods with fewer than 100 admissions have been omitted.

Fig 4 shows the annual distributions of number of diagnoses per admission stratified by the total number of previous life-time admissions that a patient has had, for all patients born since the year 2000.

## Discussion

The results of this study have demonstrated a consistent, significant, and ongoing increase in the number of diagnoses recorded for inpatients at a specialist children’s hospital over recent decades. On average, patients are associated with around three times the number of unique conditions/diagnoses per admission than at the beginning of the study. Across this period, we did not identify an increase in the average duration of hospital admissions, indicating that the increase in diagnosis number is not simply a consequence of increased patient stay in hospital.

Even when analysing the trends in diagnosing rate across different diagnosing categories from ICD-10 a similar significant trend remains, indicating that such changes are not simply due to increased utilisation or coding of ancillary services but rather that patients have a greater number of medical condition diagnoses associated with their admission. However, whether this is due to changes in patient complexity, management or referral practice cannot be determined from these data alone.

Indeed, every ICD-10 chapter (except XV – ‘Pregnancy, childbirth and the puerperium’ which has no diagnoses given that the study site has no obstetric service), were associated with an increase in coexisting diagnoses between 2000 and 2023. 19 of the 22 chapters showed a strongly increasing trend with Mann-Kendall trend test results of greater than 0.7. With few exceptions, the diagnosis rate trends for all chapters of ICD-10 diagnoses have monotonically increased year on year. Furthermore, the trends show several distinct patterns. While some areas demonstrate an increase in rates approximately linearly across the entire period (e.g., chapter VI – ‘Diseases of the nervous system’), others appear to show stepwise trends with distinct changepoints where their gradient increases by a large amount (e.g., chapter V – ‘Mental and behavioural disorders’ around 2010), while others follow different patterns or have different changepoints.

The analysis of diagnosis rates by age demonstrates similar increasing trends across all patient age groups and therefore suggests these trends are not simply a result of the average increasing patient age shown in Table 1.

The analysis of diagnosis rates by number of previous admissions also suggests that these increasing rates of diagnosis are not simply caused by few highly complex patients that are seen repeatedly. Even diagnoses from first admissions have increased over the study period. However, the greatest increases are clearly seen in patients with greater than 10 previous admissions.

There are many potential causes and implications for the increase in the rates of coexisting diagnoses across the period of this study. The key factors are likely to be a combination of changes in clinical reporting guidelines, changes in clinical diagnostic practice, and possible increased ease of recording diagnoses due to widespread use of electronic health records. However, GOSH has had versions of electronic patient records across the entire period, and all diagnoses are verified by a single clinical coding team with standard NHS guidelines for coding practices. A single comprehensive electronic patient record system was introduced in 2019 (Epic) which does align with a change in diagnosis trajectories across some analyses; however, all had well established increasing trends well before 2019, suggesting that simple recording factors are unlikely to be a major contributing factor.

The largest change in diagnostic trends across the study period was for chapter XXI – ‘Factors influencing health status and contact with health services’, which increased by 2.77 diagnoses per admission. This chapter exhibits relatively consistent diagnosis levels between 2000 and 2016, but then saw a very steep increase to approximately four times the level in four years. This chapter relates to utilisation and subsequent coding of specific types of healthcare service provision, rather than underlying medical conditions themselves and hence it is likely that at least part of such increase was driven by local policy changes rather than patient condition. Nevertheless, less severe trends are observed across almost all chapters, suggesting that much of the trend effect relates to medical conditions and cannot be explained simply by policy changes regarding coding of support service provision.

In addition to direct illustration of the apparent increasing complexity of in-patients in paediatric practice, these findings also illustrate the importance of considering the contemporaneousness of data used in retrospective research studies. This consideration is particularly important in the context of artificial intelligence and digital health applications concerned with the development of clinical support tools based on routinely acquired historical data. Naïve methods and analyses would be very likely to misinterpret the causes and implications of co-occurring diagnosis trends observed here. Further work is needed to analyse this effect while controlling for primary diagnosis complexity; however, the data suggest that recent patients can be expected to have significantly more comorbidities associated with a given condition than they would have recorded with the same apparent condition admitted 10 or 20 years ago. Any analysis, model or method that does not compensate for this may therefore incorrectly determine the significance of such comorbidities if temporal trends are not accounted for. Critically, clinical coding of diagnosis is inseparably linked to financial reimbursement for healthcare providers and is therefore the subject of strict rules [8]. However, changing rules and reimbursement models will inevitably affect these results.

The primary strength of this study is that is analyses 24 years of comparable data across a whole hospital with clinical coding performed by a dedicated team according to standard guidelines. However, these results therefore represent only a single, unique, centre and therefore further work is needed to explore if similar increasing diagnosis trends are observed across other paediatric hospitals or populations. Furthermore, the hospital studied is a world-renowned specialist tertiary referral centre recognised for its expertise in managing highly complex and severe cases, and therefore the complexity and acuity of the studied patients would be expected to be different from those seen in typical tertiary care settings.

It should be noted that there are alternative ways of assessing patient complexity, such as frailty indices, that could also have been explored. In addition, the cause of the changes in co-diagnosis rates cannot be determined from these routine retrospective data. Whilst the changes are clearly not a simple consequence of increased length of stay or use of service provision codes, it remains undetermined as to what extent such changes represent true increase in patient complexity, such as through changes in referral practice, versus changes in clinical coding practices with improved recognition of multiple comorbidities.

The findings of this study should be of interest in relation to changing patterns of morbidity, healthcare resource planning and wider use of routinely collected electronic patient record data for research purposes.
